# Inflammation Induced by Natural Neuronal Death and LPS Regulates Neural Progenitor Cell Proliferation in the Healthy Adult Brain

**DOI:** 10.1523/ENEURO.0023-20.2020

**Published:** 2020-07-02

**Authors:** Tracy A. Larson, Yekaterina Tokareva, Marianne Meritt Cole, Eliot A. Brenowitz

**Affiliations:** 1 Department of Biology, University of Virginia, Charlottesville, Virginia 22904; 2 Department of Biology, University of Washington, Seattle, Washington 98195-1800; 3 Department of Psychology, University of Washington, Seattle, Washington 98195-1525

**Keywords:** apoptosis, neural plasticity, neural progenitor cell, neuroinflammation, sex steroids, songbird

## Abstract

Inflammation is typically considered a negative response to injury or insult; however, recent advances demonstrate that inflammatory cells regulate development, plasticity, and homeostasis through anticytotoxic, progenerative responses. Here, we extend analyses of neuroinflammation to natural neurodegenerative and homeostatic states by exploiting seasonal plasticity in cytoarchitecture of the avian telencephalic song control nucleus, high vocal center [HVC (proper name)], in the songbird Gambel’s white-crowned sparrow (*Zonotrichia leucophrys gambelii*). We report that local injection of the endotoxin lipopolysaccharide into HVC of birds in both breeding (high circulating testosterone level) and nonbreeding (low circulating testosterone level) conditions increased neural progenitor cell proliferation in the nearby but distinct ventricular zone. Additionally, we found that oral administration of the anti-inflammatory drug minocycline during seasonal regression of HVC reduced microglia activation in HVC and prevented the normal proliferative response in the ventricular zone to apoptosis in HVC. Our results suggest that local neuroinflammation positively regulates neural progenitor cell proliferation and, in turn, contributes to the previously described repatterning of HVC cytoarchitecture following seasonally induced neuronal loss.

## Significance Statement

We demonstrate that natural reactive neurogenesis, or the birth of new neurons in the adult brain following non-injury-induced neuronal loss, is depressed with oral administration of the anti-inflammatory drug minocycline. Minocycline—an inhibitor of microglia activation—prevents reactive neurogenesis following seasonally induced neuronal loss in the avian telencephalic nucleus, high vocal center [HVC (proper name)]. Conversely, local inflammation, induced by microinjecting endotoxin directly into HVC, increases neural progenitor cell proliferation in the adjacent ventricular zone, which supplies HVC with new neurons. These findings contribute to the emerging role that neuroinflammatory cells play in regulating adult neurogenesis and promoting circuit homeostasis and regeneration, and establish the avian song control circuit as a useful model for future studies examining mechanisms of neuroinflammation.

## Introduction

Inflammatory responses are important for debris and pathogen clearance, tissue repair, and in some contexts, protection from future insults. Inflammation has been classically viewed as detrimental. Yet, emerging evidence indicates that inflammatory cells play critical roles in establishing tissue pattern; plasticity of cell morphology and behavior; and maintenance of cellular, tissue, and organ homeostasis (Hammond et al., 2018). The effects of inflammation depend on several factors including the following: the location and cellular heterogeneity of the tissue in which inflammation occurs (Marshall et al., 2014); the duration of the inflammatory event (Kohman and Rhodes, 2013); and the age, sex, and hormonal status of the individual (Villa et al., 2016). Consequently, a shift away from positive protective inflammatory effects underlying the processes of development, plasticity, and homeostasis toward more detrimental cytotoxic inflammation likely contributes to the pathologic consequences of many diseases including depression, Alzheimer’s disease, epilepsy, and multiple sclerosis, among others (Wynn et al., 2013).

Within the CNS, inflammatory cells can have seemingly contradictory effects on mature neuronal survival, glial support and scarring, circuit structure, neural progenitor cell (NPC) proliferation and the survival and function of new neurons (Larson, 2018). The impact of neuroinflammation on NPCs and their progeny depends on the phenotypic state of the responding cells—typically astroglia and microglia. For example, in the healthy brain, “resting” or ramified microglia play a beneficial role by phagocytosing excess NPC progeny to maintain proper neuronal number (Sierra et al., 2010). Following tissue damage, microglia become polarized and respond through a continuum of functional states. The impact of these functional states ranges between “negative” cytotoxic inflammation (Prinz and Priller, 2014) and “positive” resolution through the release of anti-inflammatory cytokines and growth factors (Hu et al., 2012). The dynamic impacts of inflammatory cells in the CNS, together with the fact that not all neurodegeneration is injury or pathogen induced, highlight the need for examining neuroinflammation within the contexts of naturally occurring neuronal death and maintenance of homeostasis in otherwise healthy adult brains.

**Figure 1.**
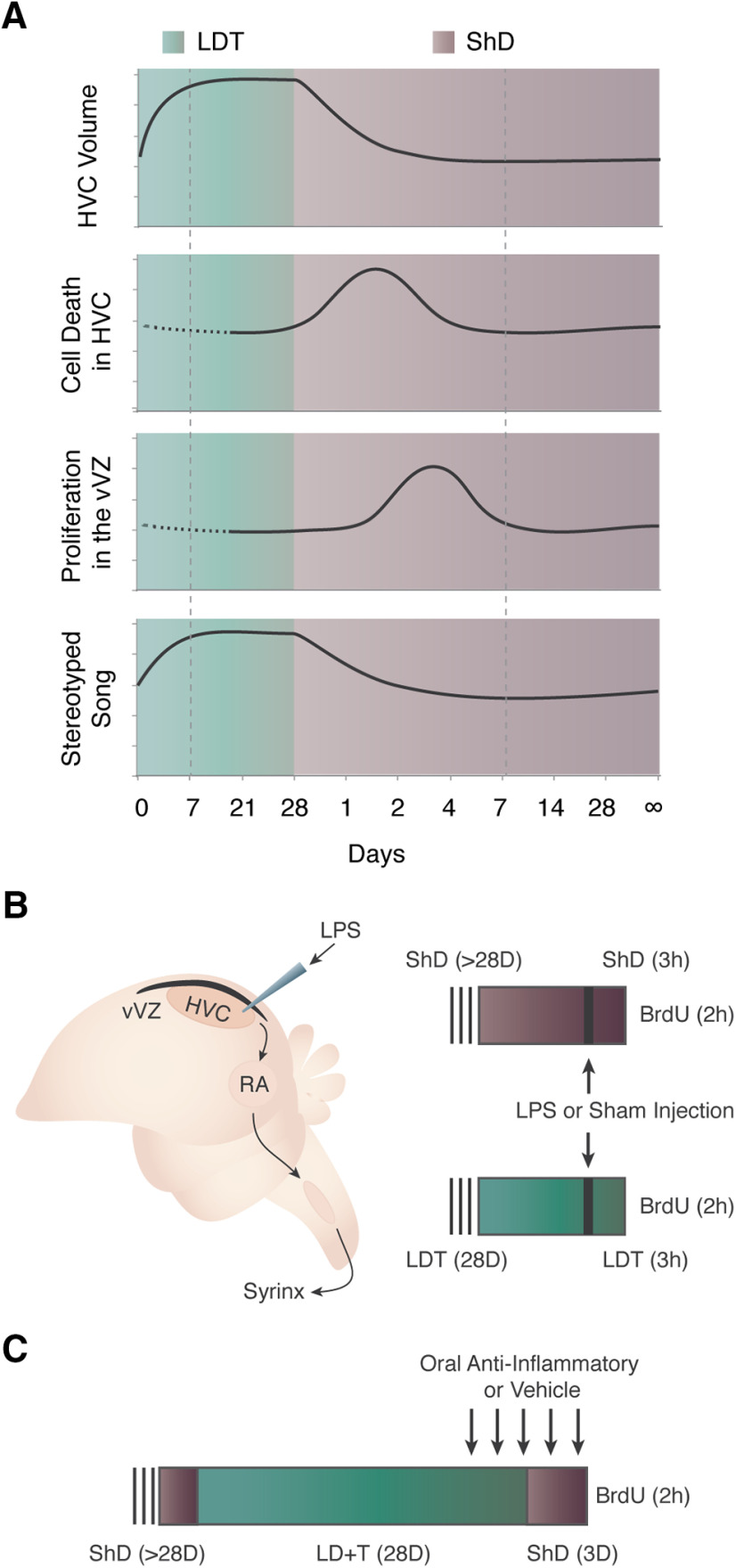
Experimental background and design. ***A***, Seasonal growth and regression of HVC and effects on song stereotypy are illustrated. As white-crowned sparrows transition from breeding (green background) to nonbreeding conditions (purple background), HVC regresses in volume due to cell death. Increased proliferation of NPCs in the vVZ (black strip dorsal to HVC) that gives rise to new HVC neurons follows temporally and is induced by HVC cell death (Larson et al., 2014). Morphology and physiology reaches homeostasis in both breeding and nonbreeding conditions (indicated by dotted box). Dotted line indicates a lack of published data for those time points. ***B***, Schematics illustrating the song production circuit and experimental design for testing the role of acute, local inflammation on NPC proliferation. The song production circuit consists of HVC (proper name), the RA, and nXIIts, nucleus para-ambiguus. Birds in nonbreeding (ShD; purple) or breeding (LDT; green) condition for 28 d received LPS through needle microinjection into HVC, while controls received sham injections. To label proliferating NPC, BrdU was administered systemically 1 h following LPS or sham injection, and 2 h before tissue harvesting. ***C***, Experimental design to test the necessity of inflammation for reactive neurogenesis following natural cell death in HVC. Birds received oral doses of the anti-inflammatory agent minocycline starting 2 d before and continuing throughout the 3 d of HVC regression after rapid transition of birds from LDT to ShD conditions. Control birds received oral doses of water. All birds were injected with BrdU to label proliferating NPC 2 h before tissue harvesting.

Seasonal regression of the avian telencephalic song nucleus high vocal center [HVC (proper name); Reiner et al., 2004] and its efferent target, the robust nucleus of the arcopallium (RA), provides a unique and valuable model to investigate inflammatory modulation of NPC proliferation during stable states and after nonpathologic neural degeneration (Fig. 1*A*). In adult male Gambel’s white-crowned sparrows (*Zonotrichia leucophrys gambelii*), HVC neuronal number changes seasonally (Smith et al., 1997). Within 1 week of breeding condition onset, HVC growth stabilizes with as many as 68,000 new neurons incorporated into the existing 100,000 mature neurons (Tramontin et al., 2000). Within 4 d of transition from breeding to nonbreeding conditions, an equal number of HVC neurons die via caspase-dependent cell death (Thompson and Brenowitz, 2008). HVC cell death increases proliferation in the adjacent, but distinct ventral ventricular zone (vVZ; Larson et al., 2014), which supplies HVC with new neurons (Scott and Lois, 2007). Proliferation in the vVZ occurs primarily through rapidly dividing radial glial neural progenitor cells (Scott and Lois, 2007), but also presumably includes neural stem cells, albeit at slower and lower rates. Both the rapid addition of new neurons and the natural reactive neurogenesis in the nearby vVZ precede a return of HVC to breeding and nonbreeding homeostatic states (Larson et al., 2014). The natural plasticity in neuron number, with accompanying changes in arborization and electrical signaling (DeVoogd and Nottebohm, 1981; Meitzen et al., 2007), contributes to a biologically relevant and plastic behavior: the production of learned song (Meitzen et al., 2009).

Because HVC neuronal loss, reactive neurogenesis, and subsequent return to homeostasis occur robustly and without prior neural insult, white-crowned sparrows are useful for examining the processes and mechanisms by which neuroinflammation modulates NPC under nonpathologic conditions in the adult brain. We report that the induction of inflammation locally within HVC rapidly increased NPC proliferation in the vVZ. Alternatively, decreasing microglia activation following natural neuronal loss in HVC reduced the reactive increase in NPC proliferation. Our work provides the first evidence for inflammatory modulation of NPC proliferation under a natural, non-injury-induced neurodegenerative state in the intact adult brain.

## Materials and Methods

*Animals.* Male Gambel’s white-crowned sparrows were collected in eastern Washington during seasonal migration. Upon capture, all birds were aged with a minimum age based on plumage coloration. Birds with juvenile plumage (brown crown) were assigned an age of 2 months. Because precise age cannot be determined in white-crowned sparrows once a bird has attained its adult plumage (black and white crown), all adult birds were assigned a minimum age of 14 months on collection. All birds were housed in indoor aviaries exposed to short-day (ShD) photoperiods (ShDs; 8 h light, 16 h dark) for at least 10 weeks to ensure that their reproductive systems had fully regressed, and that the birds would be sensitive to the stimulatory effects of transition to long day photoperiods (LDs; 20 h light, 4 h dark) and testosterone (T) treatment. Because age affects levels of cell death and neurogenesis in white-crowned sparrows (Larson et al., 2014), adult male birds were assigned semirandomly to experimental groups with age being the only factor purposely selected and balanced across groups. All experiments followed National Institutes of Health (NIH) animal use guidelines and were approved by the University of Virginia and the University of Washington Institutional Animal Care and Use Committees.

**Figure 2.**
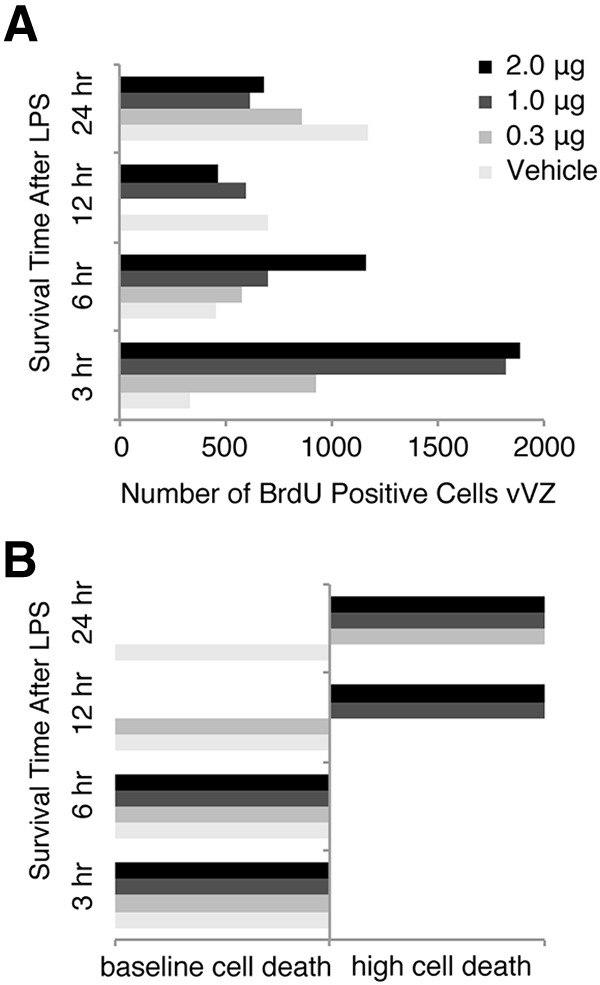
Identification of an LPS injection dose and pulse time that increases vVZ NPC proliferation but does not increase HVC cell death. ***A***, BrdU-positive cells in unilateral vVZ following injection of LPS ranging from 0 to 2 μg and a survival time of 3–24 h before tissue harvesting. NPC proliferation appeared to increase as soon as 3 h following LPS injection for all groups except saline vehicle. Saline appeared to increase the number of BrdU-positive vVZ cells by 24 h following HVC injection. Each experimental and control group, *n* = 2. ***B***, Grossly observed cell death in HVC following injection of LPS or vehicle over the time course. LPS appeared to induce cell death as soon as 12 h following LPS injection in a dose-dependent manner. By 24 h, all doses of LPS induced HVC cell death obviously visible by large numbers of Nissl-stained pyknotic cells. LPS appeared to not induce large quantities of HVC cell death with 3 and 6 h survival times. Because a dose of 1 μg of LPS induced a large increase in vVZ proliferation but no cell death after 3 h, we chose this dose and time course for our experiment.

*Experimental design.* We injected HVC with lipopolysaccharides (LPSs), a lipoglycan endotoxin from the outer membrane of Gram-negative bacteria commonly used to mimic CNS infection and induce microglia activation (Buttini et al., 1996; Hauss-Wegrzyniak et al., 1998). To identify a dose and duration that promoted inflammation but not cell death, we injected LPS of varying concentrations (0.3, 1, or 2 μg in 60 nl of 15% DMSO, 7% sodium chloride) into HVC of two birds in ShD conditions for each possible treatment. Briefly, after anesthetizing birds with isoflurane (1.5–2%), we lowered a glass micropipette into HVC lateral to the intersection of the midsagittal and transverse sinuses (0.7 mm ventral, 2.3 mm) and pressure injected LPS or vehicle (60 nl of 15% DMSO in 7% sodium chloride saline). Tissue was collected from two birds for each injected dose of LPS at each of the following survival times: 3, 6, 12, and 24 h. Upon examination of BrdU-immunolabeled and Nissl-stained tissue for vVZ proliferation and pyknotic cells, respectively (methods for staining are described below), we determined that a dose of 1 μg injected 3 h before the termination of the experiment optimally induced NPC proliferation (Fig. 2*A*) without inducing cell death beyond baseline levels (Larson et al., 2019) as detected with Nissl staining (Fig. 2*B*).

**Figure 3.**
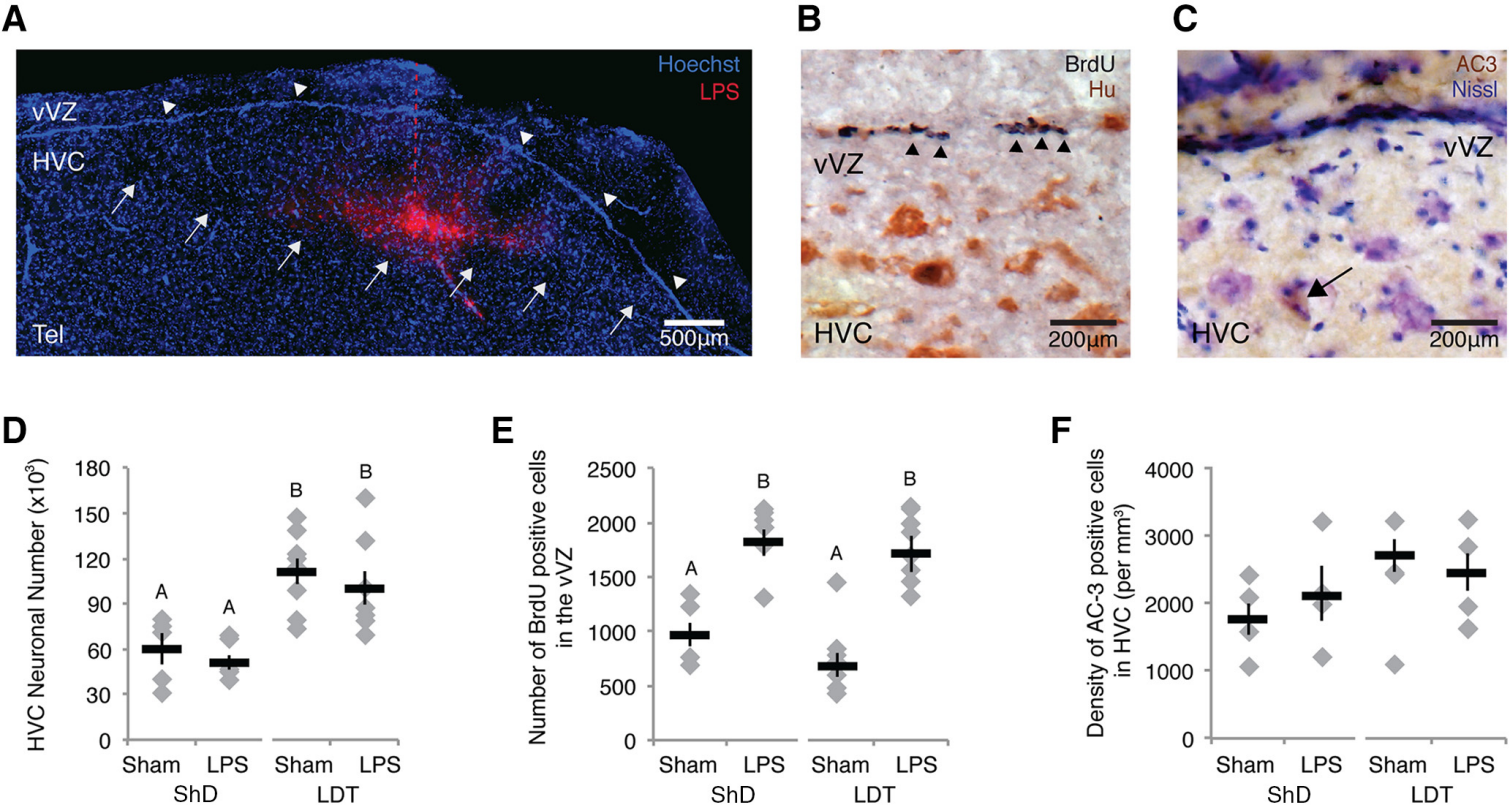
Local, acute inflammation induced a rapid increase in NPC proliferation independently of physiological condition (i.e., breeding or nonbreeding) and cell death. ***A***, Image of Alexa Fluor-conjugated LPS localization within HVC 3 h after microinjection. LPS is shown in red; Hoechst 33342, a nuclear stain, is shown in blue. White arrows indicate the ventral boarder of HVC, white arrowheads indicate the vVZ, and the red dotted line indicates the injection needle track. Tel, Telencephalon. ***B***, BrdU labeling (dark purple, indicated by black arrowheads) in the vVZ, just dorsal to HVC. Tissue was colabeled with an antibody against the neuronal antigen HuC/D to provide histologic landmarks during analyses. ***C***, Cell death within HVC as indicated by AC-3-positive immunolabeling in brown, as indicated by the black arrow. Tissue is Nissl counterstained to identify the borders of HVC. ***D***, Number of all HuC/D-positive neurons (i.e., new plus mature) in one hemisphere of HVC across experimental and control groups. The HVC neuron number was consistently lower in nonbreeding than breeding conditions. LPS did not affect the total neuronal number in breeding or nonbreeding condition birds. ***E***, Number of BrdU-positive cells in the vVZ of experimental and control groups in breeding and nonbreeding conditions. LPS increased the numbers of new cells in vVZ equally in both breeding and nonbreeding condition birds. ***F***, The density of AC-3-positive cells did not differ between LPS-injected and control birds, or between long-term breeding and long-term nonbreeding conditions. ***E***, ***F***, Different letters above error bars indicate significant differences among groups, as determined with *post hoc* Tukey’s test analyses (Table 1, ANOVA effects). All data are plotted as the mean ± SEM (black), with data for each individual bird displayed as gray diamonds.

Birds were housed in either ShD (*n* = 11) or LD with implantation of a subcutaneous 12 mm SILASTIC capsule (1.47 mm inner diameter, 1.96 mm outer diameter) filled with crystalline T (Sigma-Aldrich) above the scapula [i.e., LD and exogenous T (LDT), *n* = 18; Fig. 1*B*]. After 28 d in ShD or LDT, we injected HVC with LPS (1 μg in 60 nl of 15% DMSO, saline; ShD, *n* = 6; LDT, *n* = 9). To ensure that the end of the needle track was within HVC, four birds were injected with Life Technologies LPS Alexa Fluor 568 Conjugate (1 μg in 60 nl of 15% DMSO, saline; Thermo Fisher Scientific). Histologic analysis confirmed that the end of the needle track was in HVC of all four birds (Fig. 3*A*). Control birds (ShD, *n* = 5; LDT, *n* = 9) had the needle lowered into HVC with no injection of fluid. One hour following surgery and 2 h before tissue collection, all birds were injected intraperitoneally with BrdU (50 mg/kg; 15 mg/ml in 15% DMSO in PBS) to label proliferating NPC and their recent progeny in the vVZ.

**Table 1:** Sex steroid levels, morphometrics, and cellular changes with induction of inflammation

	ShD		LDT		ANOVA main effect(two-way)	Injection type(Sham vs LPS)	Condition (ShD vs LDT)	Injection * Condition
	Sham (*n* = 5)	LPS (*n* = 6)	Sham (*n* = 9)	LPS (*n* = 9)				
Testosterone								
Plasma levels (ng/ml)	0.07 ± 0.03^a^	0.08 ± 0.02^a^	15.80 ± 1.69^b^	17.70 ± 1.65^b^	*F*_(3,20)_ = 31.8914; *p* < 0.0001	*F* = 0.2986; *p* = 0.5908	*F* = 990.7318; *p* < 0.0001	*F* = 0.2920; *p* = 0.5949
HVC								
Unilateralvolume (mm^3^)	0.42 ± 0.05^a^	0.41 ± 0.04^a^	0.80 ± 0.05^b^	0.75 ± 0.06^b^	*F*_(3,24)_ = 10.6481; *p* < 0.0001	*F* = 0.3873; *p* = 0.5396	*F* = 30.5611; *p* < 0.0001	*F* = 0.2033; *p* < 0.0001
Neuron density(×10^3^/mm^3^)	130 ± 5^a,b^	117 ± 4^a^	129 ± 3^b^	128 ± 4^b^	*F*_(3,25)_ = 6.8570; *p* = 0.0016	*F* = 1.8998; *p* = 0.1803	*F* = 16.3837; *p* = 0.0004	*F* = 1.9168; *p* = 0.1784
Unilateral neuronnumber (×10^3^)	60 ± 10^a^	51 ± 5^a^	111 ± 9^b^	100 ± 11^b^	*F*_(3,24)_ = 5.3377; *p* = 0.0058	*F* = 0.0773; *p* = 0.7834	*F* = 15.1049; *p* = 0.0007	*F* = 0.5399; *p* = 0.4696
AC-3 cell density(per mm^3^)	1747 ± 230	2132 ± 411	2696 ± 241	2445 ± 271	*F*_(3,21)_ = 2.0696; *p* = 0.1349	*F* = 0.0432; *p* = 0.8373	*F* = 4.4773; *p* = 0.0465	*F* = 1.1176; *p* = 0.3025
Unilateral AC-3 cell number	829 ± 200^a^	946 ± 245^a^	2328 ± 292^b^	2060 ± 360^a,b^	*F*_(3,21)_ = 5.9919; *p* = 0.0041	*F* = 0.1334; *p* = 0.7186	*F* = 16.1711; *p* = 0.0006	*F* = 0.4803; *p* = 0.4803
Microglia in HVC								
Ramified	295 ± 86	231 ± 29	479 ± 38	451 ± 73	*F*_(3,18)_ = 2.6917; *p* = 0.0770	*F* = 2.1850; *p* = 0.1566	*F* = 5.4407; *p* = 0.0315	*F* = 1.0983; *p* = 0.3085
Intermediate	85 ± 30	128 ± 11	142 ± 23	139 ± 40	*F*_(3,18)_ = 0.5415; *p* = 0.660	*F* = 0.3760; *p* = 0.5474	*F* = 1.1190; *p* = 0.3041	*F* = 0.2635; *p* = 0.6139
Reactive	32 ± 12	43 ± 7	48 ± 10	51 ± 11	*F*_(3,18)_ = 0.4788; *p* = 0.7010	*F* = 0.4027; *p* = 0.5337	*F* = 1.0125; *p* = 0.3276	*F* = 0.0982; *p* = 0.7576
Total active	129 ± 41	156 ± 12	190 ± 33	190 ± 49	*F*_(3,18)_ = 0.4510; *p* = 0.7197	*F* = 0.0976; *p* = 0.7583	*F* = 1.1961; *p* = 0.2885	*F* = 0.1018; *p* = 0.7533
Total all	552 ± 108	361 ± 41	668 ± 57	641 ± 93	*F*_(3,18)_ = 2.5088; *p* = 0.0915	*F* = 1.6603; *p* = 0.2139	*F* = 5.4621; *p* = 0.0312	*F* = 0.9271; *p* = 0.3484
vVZ								
Unilateral BrdU-positive cel number	971 ± 164^a^	1819 ± 123^b^	690 ± 113^a^	1712 ± 106^b^	*F*_(3,22)_ = 22.0745; *p* < 0.0001	*F* = 54.4770; *p* < 0.0001	*F* = 2.394; *p* = 0.1412	*F* = 0.4709; *p* = 0.4997
Age								
Minimum age(month)	21 ± 2	19 ± 3	20 ± 2	17 ± 2	*F*_(3,25)_ = 0.3227; *p* = 0.8089	*F* = 0.5843, *p* = 0.4518	*F* = 0.2273, *p* = 0.6377	*F* = 0.0493, *p* = 0.8262

All values are the mean ± SEM. Superscript letters denote significant differences across treatment groups with *post hoc* Tukey’s test, only if the main effect of ANOVA was significant.

We blocked inflammation with an oral anti-inflammatory drug, minocycline, during rapid regression of HVC (Fig. 1*C*). Minocycline directly inhibits microglia transition to the activated state, and thus indirectly attenuates the innate inflammatory response through decreased TNF-α, cytokine, and interleukin production (Giuliani et al., 2005; Kobayashi et al., 2013). All birds were housed in LDT for 28 d to allow full breeding-like growth of the song control nuclei (*n* = 21; Tramontin et al., 2000). To induce regression of HVC, birds had T pellets removed after 28 d of LDT and were transitioned onto an ShD light schedule overnight (*n* = 17; Larson et al., 2014). Two days before transition (i.e., LDT at 26 d), birds began receiving a twice-daily oral dose of 30 μl of minocycline (Sigma-Aldrich; 50 mg/ml in distilled water; *n* = 10) or distilled water (*n* = 7) for 5 d. Following T pellet removal, birds were housed in ShD for 3 d. On the third day of LDT withdrawal (LDW) and 2 h before tissue collection, all birds were injected with one dose of BrdU (50 mg/ml) to label the proliferation of NPCs and their recent progeny in the VZ. As a control for off-target or basal effects of minocycline, another group of birds received oral administration of minocycline and a BrdU injection as described above but were maintained in LDT for the duration of the experiment (*n* = 4). Unfortunately, due to the required repeated handling of the birds, we were not able to obtain singing behavior in this study.

*Blood draw and hormone analysis.* Blood samples were obtained from all birds after they were killed to measure circulating T concentrations. We drew ∼250 μl of blood from the alar vein in the wing into heparinized collection tubes, immediately centrifuged the tubes, and stored the plasma at −20°C until assay. Plasma T concentrations were measured using a Testosterone Enzyme Immunoassay kit (Enzo Life Sciences). Samples with undetectable levels of T (*n* = 11) were treated as having concentrations at the minimum detection (0.03 ng/ml for ShD birds) or maximum detection (20.00 ng/ml for LDT birds) limit for statistical analyses. Intra-assay and interassay coefficients of variation were 0.59–41.25% and 10.00%, respectively.

*Tissue collection and processing.* Five to 7 h after lights-on, birds were deeply anesthetized with isoflurane (4%). Within 2 min, brains were removed and bisected at the midline. One randomly chosen hemisphere was frozen on dry ice for histology and stored at −80°C until cryosectioning. Each frozen brain was sectioned in the coronal plane at 40 μm on a cryostat with each section thaw mounted serially. Every third slide was Nissl stained with thionin, and the remaining slides were stored at −80°C until immunolabeling. The other half of each brain was used for piloting a different experiment not included here; thus, all histologic measurements described below are reported as unilateral counts or densities. No significant effects of right versus left hemisphere were identified within experimental groups, and so all data were pooled for each experimental group.

*Immunohistochemistry analyses.* To confirm localization of LPS (Fig. 3*A*), we fixed slides of mounted tissue in 4% paraformaldehyde (PFA) and counterstained tissue with the nuclear marker Hoechst 33342 (0.2 mg/ml in distilled H_2_O) for 20 min. Slides were rinsed in PBS and mounted with anti-fade mounting medium (1% n-propyl-gallate, 90% glycerol in PBS).

To visualize BrdU-labeled cells (Fig. 3*B*), mounted sections were fixed in 4% PFA, rinsed with PBS with 0.5% DMSO and 0.5% Triton-X (PDTX; pH 7.4), dipped in distilled water, incubated with 2N HCl at 37°C for 30 min, and rinsed with PDTX. After blocking in 5% heat-inactivated goat serum (Vector Laboratories) for 1 h, slides were incubated serially with rat anti-BrdU (1:200; catalog #MCA2060, Bio-Rad) and Invitrogen mouse anti-HuC (1:100; Thermo Fisher Scientific) antibodies. HuC is a neuron-specific antigen expressed in both immature and fully differentiated neurons (Barami et al., 1995). Labeling was visualized with diaminobenzidine (DAB) staining following incubation with biotinylated goat anti-rat and anti-mouse IgG secondary antibodies (1:200; Vector Laboratories) and amplification with avidin–biotin peroxidase complex (ABC kit, Vector Laboratories). To obtain a purple precipitate for the BrdU antigen, we used DAB-nickel stain (0.05% DAB, 0.05% nickel ammonium sulfate, and 0.015% H_2_O_2_ in PBS). BrdU-positive cells in the vVZ were counted from the hillock of the VZ medial to HVC, out to the most lateral extension of the vVZ in all sections that exhibited arching of the VZ (Scott and Lois, 2007; Larson et al., 2014).

Activated caspase-3 (AC-3) immunolabeling was used to identify cells undergoing apoptosis (Fig. 3*C*; Srinivasan et al., 1998). Sections were blocked with 5% heat-inactivated goat serum, incubated with rabbit anti-AC-3 antibody (1:400; catalog #13847, Abcam), and then incubated with biotinylated goat anti-rabbit IgG secondary antibody (1:200; Vector Laboratories; Larson et al., 2014). DAB staining was performed following amplification with the ABC kit (Vector Laboratories). Sections were lightly counterstained with thionin, a Nissl stain. AC-3-positive cells were counted from at least four HVC sections per bird using a previously described sampling protocol (Larson et al., 2014).

**Figure 4.**
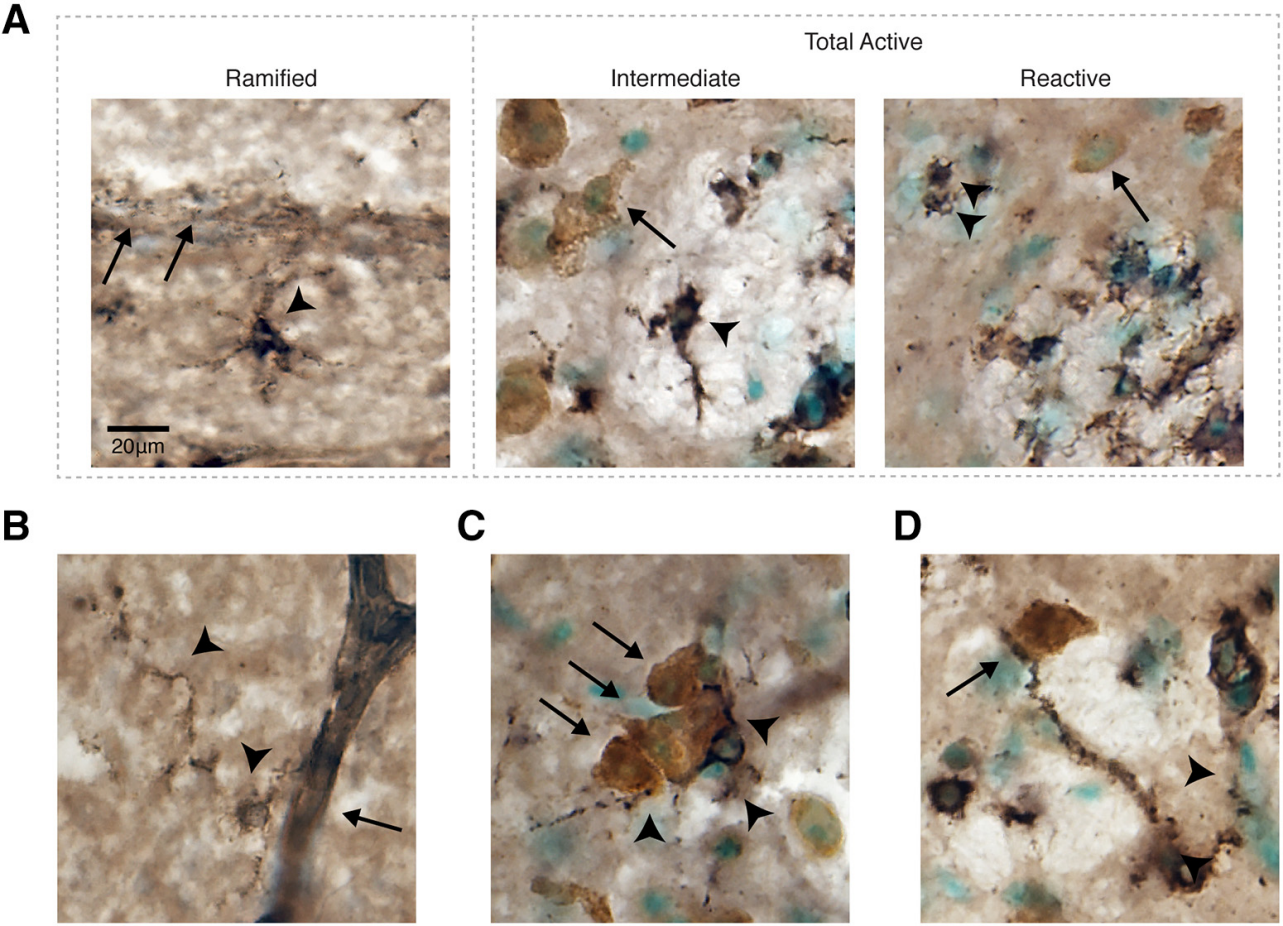
Representative images of microglia with different morphologies observed within HVC. ***A–D***, Microglia are stained with RCA-I (black), and neurons are immunolabeled for neuronal antigen HuC/D (brown). Nuclei are counterstained with methyl green (green). ***A***, Three morphologies of microglia quantified in HVC (within dotted box). Ramified microglia (arrowhead) in close proximity to the vVZ (arrows; farthest left image). Active microglia including intermediate microglia (arrowhead; middle image) and reactive microglia (arrowhead; farthest right image) with commingling with neurons (arrows). ***B***, Ramified microglia (arrowhead) in HVC contacting the vasculature (arrow). ***C***, Ramified microglia (arrowhead) with processes intermingling with neuronal soma (arrows). ***D***, Microglia with processes in contact with neuronal soma (arrow) and a non-neuronal cell (arrowhead).

Microglia were detected with biotinylated *Ricinus communis* Agglutinin I (RCA-I; 1:250; catalog #B-1085, Vector Laboratories; Mannoji et al., 1986) after 4% PFA for 20 min, 15% MeOH in PBS for 15 min, PBS rinses, and 5% heat-inactivated goat serum for 1 h. To visualize labeling, DAB-nickel staining was performed as above following amplification of the biotinylated lectin with the ABC Kit (Vector Laboratories). Neurons were labeled with the mouse anti-HuC antibody and detected with a biotinylated goat-anti-mouse antibody and DAB, as described above. To counterstain tissue, slides were briefly dipped in distilled water, incubated in methyl green (0.5% ethyl violet in 0.1 m sodium acetate, pH 4.2) at 60°C for 5 min, and rinsed in distilled water until the runoff was clear. Microglia were quantified within the full extent of HVC based on morphologic characteristics previously described (Perez-Pouchoulen et al., 2015). Categories included the following: (1) resting or ramified microglia with three or more thin processes; (2) intermediate microglia with one or two thick processes; and (3) reactive microglia with stout, thick processes or round/amoeboid morphology (Fig. 4).

All immunohistochemistry was performed in batches of up to 28 slides with tissue from randomly chosen birds. Each batch included one positive and one negative control slide, which, if it failed, forced the removal of the entire batch from subsequent quantification. Thus, not all measurements include data from all experimental birds. All measurements were made blind to experimental group. All cell counts were adjusted using the Konigsmark formula number four for cell splitting (Konigsmark, 1970).

*Morphometric measurements.* The volume of HVC was determined by tracing the borders of the nuclei identified in thionin-stained tissue onto paper and quantifying the area of the digitally scanned tracing using ImageJ Software (version 1.46; NIH; http://rsb.info.nih.gov/ij/). Total volumes were calculated from areas of the tracings using the formula for a truncated cone (Tramontin et al., 1998). All HuC-positive neurons that fell within an ocular grid (0.0156 mm^2^ at 400×) randomly positioned within every section through the full extent of HVC were counted to calculate HVC neuronal density. Neuronal number was obtained by multiplying neuronal density for each bird by that bird’s unilateral HVC volume.

*Statistical analyses.* Comparisons of LPS were made with two-way ANOVAs with breeding condition and treatment as the factor, whereas minocycline comparisons were made with one-way ANOVA with treatment as the factor. For microglia cell types (ramified, intermediate, and reactive), multivariate ANOVAs (MANOVAs) were performed with bird, breeding condition, and treatment as factors for the LPS study, and bird and experimental conditions as the factor for the minocycline study. For both datasets, the sphericity tests reported probabilities of <0.05 (0.0002 and 0.0185, respectively). Student’s *t* tests were performed to compare data across breeding and nonbreeding, and regressed condition with data from minocycline-treated and water-treated birds in LDW condition pooled. *Post hoc* Tukey’s tests were performed only if the overall main effect of the ANOVA was significant. Results of the *post hoc* Tukey’s test analyses are denoted by superscript letters in Tables 1 and 2 and capitalized letters in graphs. All statistical analyses were made using JMP version 8.0.1 (SAS) with an α level of 0.05. All data are presented as the mean ± SEM.

**Table 2:** Sex steroid levels, morphometrics, and cellular changes with inhibition of inflammation

	LDT		LDW	ANOVA main effect (one-way)
	Minocycline (*n* = 4)	Vehicle (*n* = 7)	Minocycline (*n* = 10)	
Testosterone				
Plasma levels (ng/ml)	10.64 ± 3.15^a^	0.45 ± 0.07^a^	0.85 ± 0.18^a^	*F*_(2,16)_ = 20.7571; *p* < 0.0001
HVC	* *	* *	* *	* *
Unilateral volume (mm^3^)	0.71 ± 0.03^a^	0.58 ± 0.01^a,b^	0.47 ± 0.04^b^	*F*_(2,17)_ = 12.8758; *p* = 0.0004
Neuron density (×10^3^/mm^3^)	133 ± 3	139 ± 3	139 ± 3	*F*_(2,16)_ = 0.9627; *p* = 0.4029
Unilateral neuron number (×10^3^)	94 ± 3^a^	80 ± 2^b^	61 ± 3^c^	*F*_(2,16)_ = 29.6698; *p* < 0.0001
AC-3 cell density (per mm^3^)	3188 ± 573^a^	8600 ± 561^b^	8941 ± 696^b^	*F*_(2,17)_ = 15.1670; *p* = 0.0002
Unilateral AC-3 cell number	2222 ± 538^a^	5033 ± 380^b^	4108 ± 153^b^	*F*_(2,16)_ = 20.3012; *p* < 0.0001
Microglia in HVC				
Ramified	237 ± 72	463 ± 72	355 ± 55	*F*_(2,9)_ = 2.5743; *p* = 0.1306
Intermediate	63 ± 27^a^	218 ± 26^b^	71 ± 12^a^	*F*_(2,9)_ = 17.4903; *p* = 0.0008
Reactive	61 ± 4	108 ± 55	40 ± 20	*F*_(2,9)_ = 1.0589; *p* = 0.3864
Total active	124 ± 27^a^	326 ± 60^b^	111 ± 21^a^	*F*_(2,9)_ = 9.6118; *p* = 0.0058
Total all	361 ± 100^a^	789 ± 67^b^	465 ± 43^a^	*F*_(2,9)_ = 11.2252; *p* = 0.0036
vVZ				* *
Unilateral BrdU-positive cell number	747 ± 54^a^	1513 ± 125^b^	751 ± 147^a^	*F*_(2,16)_ = 9.3280; *p* = 0.0021
Age	* *	* *	* *	* *
Minimum age (months)	22 ± 3	22 ± 2	21 ± 2	*F*_(2,18)_ = 0.1321; *p* = 0.8771

All values are the mean ± SEM. Superscript letters denote significant differences across treatment groups with *post hoc* Tukey’s test, only if the main effect of ANOVA was significant.

## Results

### Acute HVC inflammation rapidly increases vVZ proliferation regardless of hormonal state

Given that HVC neuronal cell death induces reactive neurogenesis (Larson et al., 2014) and that neuronal death initiates inflammatory responses in mammals (Ekdahl, 2012; Ritzel et al., 2013) and fish (Kizil et al., 2012; Kyritsis et al., 2012), we hypothesized that inflammation following HVC neuronal death regulates reactive NPC proliferation in the vVZ. To experimentally dissociate neuronal death in HVC from any putative inflammatory response, we induced local inflammation response by injecting a bacterial coat protein, LPS (1 μg; Fig. 2, dosage titration; Andersson et al., 1992; Buttini et al., 1996) into HVC of birds exposed to photoperiod and hormonal manipulations to induce a breeding (i.e., LDT) or nonbreeding-like (i.e., ShD) physiological condition (see Materials and Methods). We confirmed discrete localization of injected Alexa Fluor 568-conjugated LPS within HVC (Fig. 3*A*). We also confirmed that hormonal and light manipulations induced physiological and morphologic states typical of breeding and nonbreeding conditions. Consistent with previous reports (Smith et al., 1997; Tramontin et al., 2000), systemic testosterone (T) plasma levels, HVC volume, and HVC neuronal number were all significantly higher in breeding condition compared with nonbreeding condition (Student’s *t* test, *p* < 0.001, each; Fig. 3*D*, Table 1 for data and statistics).

To test whether local acute inflammation could rapidly elicit NPC proliferation in the vVZ, we quantified BrdU incorporation within proliferating NPCs and their recent progeny over 2 h, beginning 1 h after LPS or sham injections in HVC (Fig. 3*B*,*E*). Moreover, because estrogen, a metabolite of T, modulates neuroinflammatory responses and generally promotes neuroprotection (for review, see Larson, 2018), we compared the effects of LPS-induced inflammation in birds maintained in both breeding (LDT 28 d) and nonbreeding (ShD) conditions. In stable, long-term nonbreeding condition birds, the number of BrdU-positive cells in the vVZ was significantly higher following LPS injection compared with sham injections (*post hoc* Tukey’s test, *p* = 0.0018; Fig. 3*E*, Table 1, data and ANOVA effects). Likewise, in the stable breeding condition, LPS microinjection increased the number of BrdU-positive cells in the vVZ compared with sham injection (*post hoc* Tukey’s test, *p* < 0.0001; Table 1, Fig. 3*E*). Contrary to prediction, the number of BrdU-positive cells in the vVZ did not differ between stable nonbreeding (i.e., low T) and breeding (high T) conditions following LPS injection (*post hoc* Tukey’s test, *p* = 0.9196) or sham injection (*post hoc* Tukey’s test, *p* = 0.4687; Table 1, Fig. 3*E*). Together, these data indicate that LPS rapidly (within 3 h) increases NPC proliferation under both breeding and nonbreeding conditions, and this inflammatory-mediated proliferation of NPCs was not influenced by long-term elevation of circulating sex steroids.

To exclude the possibility that differential vVZ proliferation resulted from nonspecific cell death induced by LPS (Fig. 2; Semmler et al., 2005) or possible differences in HVC apoptosis under stable breeding and nonbreeding conditions (Kirn et al., 1994; but see Larson et al., 2014), we quantified apoptosis in HVC by immunohistochemistry for AC-3 (Fig. 3*C*,*F*; Larson et al., 2014). We found no differences in the density of AC-3-positive cells between sham-injected and LPS-injected groups (Table 1, Fig. 3*F*, data and statistics). Moreover, we found no significant difference in the density of apoptotic cells in HVC between long-term breeding and nonbreeding conditions (Table 1, Fig. 3*F*). These data confirm that differences in vVZ proliferation were not due to nonspecific changes associated with cell death or season, but rather resulted from a rapid response to local LPS microinjection.

### Local LPS injection does not rapidly induce microglia change to activated morphology

**Figure 5.**
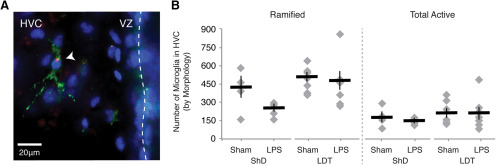
Microglial response to microinjection LPS into HVC. ***A***, A microglial cell labeled with RCA-I (green) in HVC near the vVZ (dotted line). The presence of fluorophore-conjugated LPS (red) puncta within the cell body of the microglia is suggestive of endocytosis of the LPS by microglia. Cell nuclei stained with Hoechst 33342 are shown in blue. ***B***, Number of RCA-I-labeled microglia as distinguished by morphology in HVC of one hemisphere (see also Table 1).

Given that microglia are a major cell type orchestrating inflammatory responses within the CNS following endotoxin-induced and injury-induced neuronal loss, we asked whether microglia mediated the effects of LPS in HVC on reactive neurogenesis. Following Alexa Fluor 568-conjugated LPS injection into HVC, we identified microglia present in HVC that colabeled with puncta of LPS (Fig. 5*A*). The appearance of LPS puncta in close proximity to the soma of microglia within HVC suggested that microglia were endocytosing and responding to the LPS in the avian brain. Quantification of all RCA-1-labeled microglia within HVC following LPS stimulation revealed no significant differences in the number of microglia, regardless of morphology, between LPS -injected and sham-injected groups (data and ANOVA are in Table 1, Fig. 5*B*); the MANOVA for effects of experimental conditions on microglia morphology types was not significant (between subjects: *F* = 0.4181, *p* = 0.0915; within subjects: Wilks’ λ, *p* = 0.2193). Our data indicate that neither microglia activation (as determined here by morphology rather than microglial behavior) nor proliferation occurred within 3 h of LPS administration. Thus, our data suggest that if microglia are involved in mediating the initial phases of an LPS-induced inflammatory response (as would be predicted; Buttini et al., 1996; Hauss-Wegrzyniak et al., 1998), then microglia could induce NPC proliferation without a change in morphology. Alternatively, the effects of local inflammation could be mediated, in part or entirely, through a yet undetermined cell type such as astroglia. We also found no significant differences in numbers of microglia, regardless of microglial morphology or LPS treatment, between breeding and nonbreeding conditions, suggesting that chronic elevation of sex steroids does not alter baseline microglia numbers or response to LPS.

### Microglia activation is necessary for reactive neurogenesis following seasonally induced neurodegeneration

**Figure 6.**
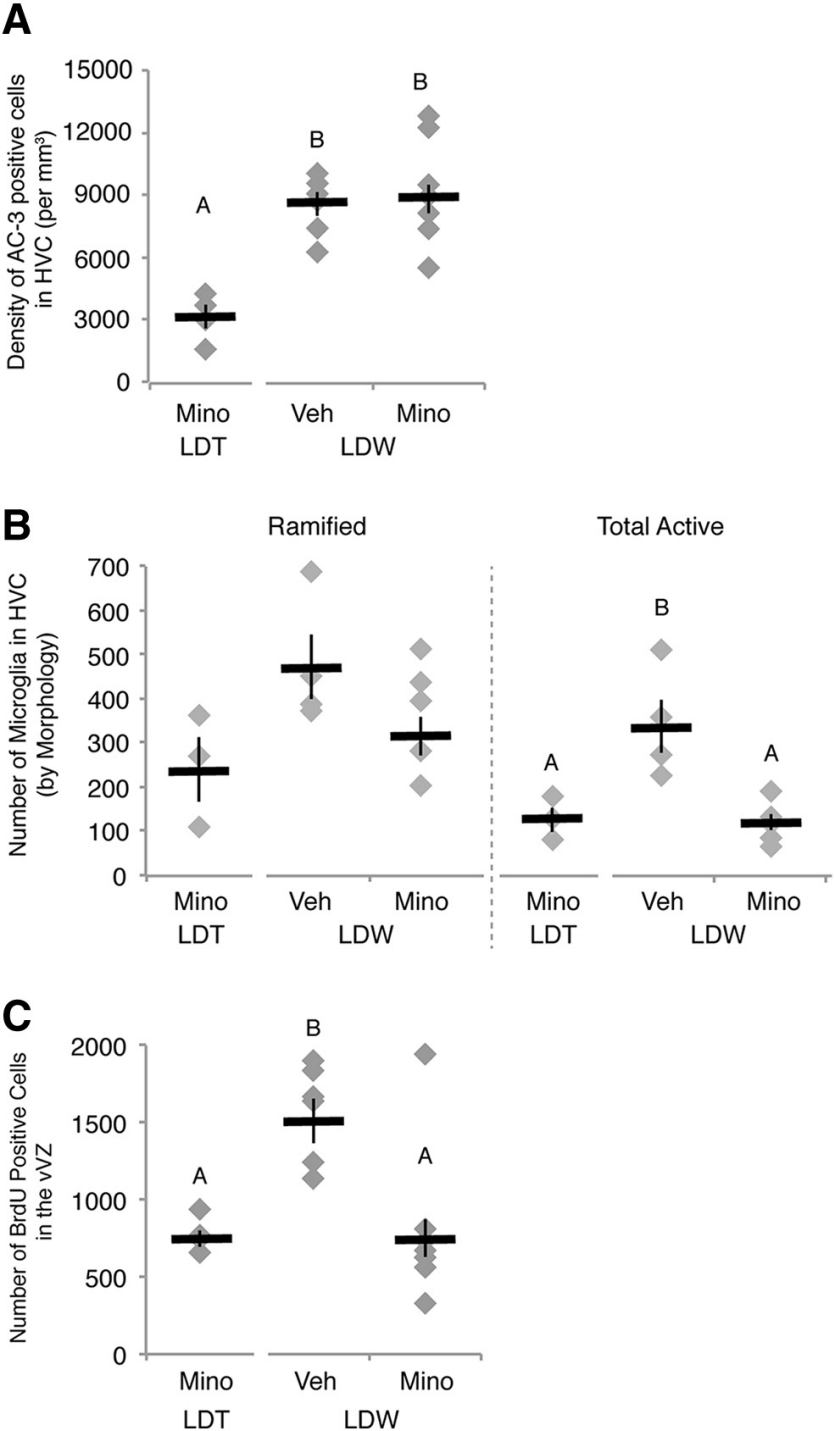
Systemic administration of minocycline (Mino) prevented natural, reactive NPC proliferation following HVC neuronal loss. ***A***, Density of apoptotic cells in HVC. The number of dying cells in HVC as shown by AC-3-positive labeling increased in birds transitioned from breeding to nonbreeding conditions and was not altered by minocycline administration. ***B***, Number of RCA-I-labeled microglia as distinguished by morphology in HVC of one hemisphere (see also Fig. 4, Table 2). The combined number of microglia with activated morphology (intermediate and reactive) was upregulated during regression [i.e., LDW with vehicle (Veh) administration (LDW-V)] when compared with breeding condition birds [i.e., LDT with vehicle (LDT-V)]. Minocycline reduced the activation of microglia during periods of neuronal loss in HVC in LDW birds (i.e., LDW-M). ***C***, The number of BrdU-positive cells in the vVZ was greater in vehicle control birds transitioned from LDT to ShD, as previously reported (Larson et al., 2014), than birds maintained in LDT. Administration of minocycline prevented reactive NPC proliferation in the vVZ during active HVC regression, suggesting that inflammation is necessary for reactive neurogenesis. ***A–C***, Letters above bars indicate significant differences among groups (ANOVA, *post hoc* Tukey’s test). Different letters above error bars indicate significant differences among groups as determined with *post hoc* Tukey’s test analyses (but see Table 2 for ANOVA effects). All data are plotted as the mean ± SEM (black), with individual data values displayed as gray diamonds.

The impact of inflammation on NPC behavior in the adult brain under non-injury-induced neural degenerative conditions is unknown. Thus, we tested whether or not inflammation is necessary for reactive neurogenesis during the transition from breeding to nonbreeding conditions, when there is extensive death of mature neurons (Fig. 1*A*; Larson et al., 2014). We inhibited putative neural inflammation with oral doses of anti-inflammatory minocycline—a selective inhibitor of microglial polarization from the ramified state (Kobayashi et al., 2013)—throughout the duration of HVC regression. We first confirmed that transition from breeding to nonbreeding conditions (i.e., LDW) induced HVC regression. Consistent with previous reports (Thompson et al., 2007; Larson et al., 2014), we found that transition to LDW decreased plasma T levels (Student’s *t* test, *p* < 0.0001; Table 2 for data), HVC volume (Student’s *t* test, *p* = 0.0016; Table 2), and HVC neuronal number (Student’s *t* test, *p* = 0.0117; Table 2) compared with values from breeding condition birds. Furthermore, as expected (Larson et al., 2014), the density and total number of apoptotic neurons were elevated in HVC of all birds transitioned to nonbreeding condition (i.e., minocycline-treated and vehicle-treated birds), compared with birds maintained in breeding condition (Student’s *t* test, *p* < 0.001 and *p* = 0.0003, for AC-3-positive cell density and total number, respectively; Fig. 6*A*, Table 2). These data confirm that photoperiod and T manipulations reproduced seasonal-like neuronal death and regression of HVC regression.

We also confirmed that minocycline prevented an anticipated upregulation of microglial activation and possible microglial proliferation with increased cell death in HVC. We found that minocycline prevented an increase in the total number of microglia and the number of activated microglia when compared with vehicle controls with one-way ANOVA (*post hoc* Tukey’s test, *p* = 0.0115 and *p* = 0.0069, respectively; Fig. 6*B*, Table 2 for data and main effect of ANOVA). MANOVA for the three microglia morphology types further supported ANOVA results with experimental conditions having a significant effect on microglia numbers (between subjects: *F* = 2.4945, *p* = 0.0036; within subjects: Wilk’s λ, *p* = 0.3857). Moreover, minocycline administration during HVC regression (LDW) reduced the total number of microglia and the number of activated microglia to levels observed in HVC of birds in stable breeding condition (*post hoc* Tukey’s test, *p* = 0.5266 and *p* = 0.9747, respectively; Fig. 6*B*, Table 2). The lower number of activated microglia with minocycline administration compared with vehicle was primarily driven by a decrease in the number of microglia with intermediate morphology (*post hoc* Tukey’s test, *p* = 0.0013; Table 2). These data confirm that minocycline inhibits microglia activation in HVC of birds and suggests that any effects of minocycline treatment on NPC proliferation would be mediated, at least in part, through the activation of microglia.

We tested the necessity of microglial activation during HVC regression for subsequent reactive neurogenesis in the nearby vVZ and found that the number of BrdU-positive cells in the vVZ was reduced in minocycline-administered birds compared with birds receiving water vehicle (*post hoc* Tukey’s test, *p* = 0.0026; Table 2, Fig. 6*C*). The density and total number of apoptotic cells in HVC were not significantly different between minocycline-treated and vehicle-treated birds (*post hoc* Tukey’s test, *p* = 0.8860 and *p* = 0.1727, respectively; Table 2, Fig. 6*A*). Moreover, the total number of neurons in HVC of birds transitioned to nonbreeding conditions decreased equivalently in minocycline-treated and vehicle-treated birds (*post hoc* Tukey’s test, *p* = 0.3279; Table 2). Together, these data demonstrate that inflammation is necessary for the reactive neurogenesis in vVZ induced by the death of mature HVC neurons, but that inflammation itself does not reciprocally contribute to secondary HVC neuronal loss during natural regression.

To rule out possible systemic effects of minocycline (Strahan et al., 2017), we administered minocycline to an additional group of birds maintained in long-term breeding conditions (i.e., 28 d). BrdU-positive cell numbers in the vVZ of minocycline–LDT-treated birds did not differ from those maintained in LDT for 28 d without any treatment (data from Larson et al., 2014), or sham injected while in LDT (one-way ANOVA, *F*_(2,14)_ = 0.2696, *p* = 0.7695). Moreover, the number of HVC neurons labeled with the neuronal marker HuC/D did not differ between these treatment groups (one-way ANOVA, *F*_(2,14)_ = 1.0515, *p* = 0.3755). The number of apoptotic cells in HVC also did not differ between minocycline-treated and vehicle-treated LDT birds (Student’s *t* test, *p* = 0.3616). Effects of minocycline on HVC cell death across studies were not tested because the AC-3 antibody used for quantification of AC-3-positive cells in the 28D LDT birds from the prior study (Larson et al., 2014) was discontinued by the vendor (Tables 1, 2, AC-3 cell counts of current study with alternative AC-3 antibody).

## Discussion

How classically defined “inflammatory cells” contribute to the establishment and maintenance of cell populations, tissue structure, and ultimately organismal behavior is an important, yet understudied, question. The seasonally plastic avian song circuit provides a unique model for identifying the cellular and molecular mechanisms underlying not only normal neuronal turnover, but also changes associated with non-injury-induced neural degeneration. Here, we demonstrate that acute inflammation induced locally within HVC, even in the absence of cell death, is sufficient to promote NPC proliferation in the vVZ, adjacent to but outside HVC. We also show that inflammation is necessary for the increase in NPC proliferation following seasonally induced HVC neuronal loss, and that microglia are very likely to be involved. Our work is the first to suggest that neuroinflammatory processes modulate NPC proliferation during natural reactive neurogenesis, and provides a tractable model for future investigations of the cell types and mechanisms linking natural neuronal apoptosis, compensatory neurogenesis, and return to homeostasis.

### Local neuroinflammation drives vVZ NPC proliferation

We found that acute LPS exposure within HVC rapidly increased NPC proliferation in the vVZ, which supplies HVC with new neurons (Scott and Lois, 2007). The effects of LPS microinjection within HVC on NPC proliferation did not result from local cell death, which is, itself, known to alter NPC proliferation (Lee et al., 2005; Semmler et al., 2005). The LPS-dependent increase in NPC proliferation that we observed is consistent with some, but not all, reports in mammals. In rats, the injection of 15 μg of LPS into the striatum increased neurogenesis after 2 weeks to an extent comparable to that induced by middle cerebral artery occlusion (Chapman et al., 2015). We found an increase in NPC proliferation 3 h after the injection of 1 μg of LPS that was similar to the increase induced by cell death during HVC regression (Larson et al., 2014). Our results, however, are inconsistent with reports of decreased proliferation 5–24 h after intraperitoneal LPS administration in rodents (Vallières et al., 2002; Fujioka and Akema, 2010) and reports of decreased neurogenesis 28 d after intraperitoneal infusion of LPS in rodents (Ekdahl et al., 2003). The contradictions in proliferative response between these studies and our studies likely reflect differences in the injections sites of LPS, the time course of LPS exposure, possible types of inflammatory responses mounted, the model organism tested, or some combination of these factors (Ekdahl et al., 2003; Semmler et al., 2005; Zhao et al., 2019). Systemic inflammation induced with intraperitoneal LPS impacts proliferation and neurogenesis though a systemic cytotoxic proinflammatory response (Kohman and Rhodes, 2013). Alternatively, LPS injection directly into the brain may promote an anticytotoxic, proneurogenic response through a Toll-like receptor 4-mediated enhancement of interferon-β (Jacobs and Ignarro, 2001) and suppression of proinflammatory nuclear factor-κB activity (Vartanian et al., 2011), which together reduce inflammatory factor secretion and inflammatory cell infiltration (Kuo et al., 2016).

We also tested the role of inflammation as the signal of HVC cell death status to the vVZ NPCs in the otherwise healthy adult brain. We found that the anti-inflammatory drug minocycline prevented natural reactive neurogenesis on rapid HVC regression, indicating that inflammation is necessary for HVC apoptosis to drive increased NPC proliferation. Our findings are consistent with previous reports that microglial activation is necessary for reactive neurogenesis following an ischemic event (Kim et al., 2009), but also highlight a novel role for inflammatory glia in modulating NPC behavior following normally occurring neuronal loss in the otherwise healthy adult brain.

### Interaction between breeding condition physiology and neuroinflammation

Not only do activity, stress, and other physiological cues affect the immunogenicity of an apoptotic cell, but these factors similarly act directly on the inflammatory cells to alter their behavior. For example, following mechanical injury to the zebra finch telencephalon, activated astroglia initially promote a proinflammatory-like response through expression of the cytokines interleukin-1β and interleukin-6 increases (Duncan and Saldanha, 2011), but subsequently “switch” to a more neuroprotective response by 20 h through increased expression of aromatase, the enzyme that facilitates estradiol synthesis from T (Duncan and Saldanha, 2011). Likewise, peripheral LPS administration in zebra finches increases transcription of aromatase in the telencephalon by 24 h postinjection (Pedersen et al., 2017b). This increase in LPS-induced aromatase expression is reduced with central administration of the anti-inflammatory drug indomethacin (Pedersen et al., 2017a). In mammals, both systemic and glial-derived elevations of estradiol reduce LPS-induced proinflammatory cytokine production by microglia (Vegeto et al., 2001) and astroglia (Loram et al., 2012). Thus, we had anticipated that elevated systemic T would attenuate microglial activation and potentiate a proneurogenic response. Nevertheless, we found no interaction effects between sex steroid levels and minocycline or LPS administration on microglia number or NPC proliferation. Our results are likely due to the long-term, rather than acute, elevation of T and the localization of endotoxin stimulation. Together, these data suggest that inflammatory cell responses vary depending on the homeostatic state of the immediate environment.

### Immunogenic apoptosis occurs in HVC following natural regression

We show a significant elevation in the number of activated microglia corresponding to increased apoptosis induced in HVC by the seasonal decrease of plasma T levels. Treating birds with the anti-inflammatory agent minocycline, however, did not reduce the number of neurons or apoptotic cells in HVC. In contrast with secondary cell death caused by activated microglia following ischemia (Tobin et al., 2014), our observation suggests that total neuronal loss in HVC during transition from breeding to nonbreeding conditions is not compounded by microglial involvement in the healthy adult avian brain. The general involvement of microglia following an increase in activated caspase-3-positive cell number in HVC might initially seem to conflict with findings that caspase-3-mediated apoptosis is typically nonimmunogenic (Fink and Cookson, 2005). Yet, caspase-3 activation is likely not the sole caspase involved in HVC seasonal neuronal loss: inhibition of caspases 1, 2, and 9, in addition to caspase-3, is necessary to prevent cell death during HVC regression and subsequent reactive neurogenesis (Thompson and Brenowitz, 2008; Larson et al., 2014). Thus, seasonally induced neuronal loss in HVC is likely not entirely mediated by caspase-3 signaling, and other caspases might be responsible for the immunogenic activation of microglia and resulting reactive neurogenesis.

### Cellular mediators of the local inflammatory response

At first glance, our finding that LPS did not induce morphologic hallmarks of microglial activation might suggest that microglia do not contribute to the inflammatory-mediated increase of NPC proliferation. Nevertheless, given that microglial retraction of processes begins at 3 h and peaks at ∼8 h following peripheral LPS administration (Buttini et al., 1996), and that microglia can secrete cytokines in their “resting” or ramified state (for review, see Cherry et al., 2014), it may not be surprising that we did not observe a significant increase in the number of activated microglia 3 h after LPS injection into HVC. Although microglia are the dominant immunogenic cells within the brain and are well documented to respond robustly to LPS (Buttini et al., 1996; Hauss-Wegrzyniak et al., 1998), astrocytes can also become reactive and exhibit immune-like responses following neural insults including LPS stimulation (Wu et al., 1998). Our data support a model in which local inflammation stimulates NPC proliferation, though we cannot yet definitively assign these functions to one or the other cell type. Development of new reagents and tools for the avian system should permit such assessment in future efforts.

### Role of local inflammatory cells in homeostasis

The impact of inflammation on cell behavior and tissue integrity has been examined primarily within the context of response to injury or endotoxin-induced insults. Yet, classically defined immune cells have roles beyond immune responses: macrophages, and by extension microglia, can act outside their classic inflammatory roles to regulate metabolism, vascularization, fluid balance, and development of tissue pattern (Wynn et al., 2013). For example, in tissues with underlying branching morphology, such as lung, kidney, mammary gland, and neural tissues, macrophages regulate patterning through the deposition of the surrounding collagen matrix and vasculature (for review, see Wood and Martin, 2017). Macrophages also provide a signaling relay between pigment cell types during larval-to-adult repatterning of the zebrafish pigment pattern (Eom and Parichy, 2017). Likewise, we found that the activation of local inflammatory cells promotes the previously described repatterning of HVC cytoarchitecture to the nonbreeding state through the modulation of NPC proliferation following natural neuronal loss in the adult avian forebrain (Larson et al., 2014). Our results are additionally concordant with the observation of microglia behavior during the development of the rat brain (Shigemoto-Mogami et al., 2014) in which activated microglia accumulate in the subventricular zone—a forebrain neurogenic niche in the mammalian brain—and secrete proinflammatory cytokines that stimulate early postnatal neurogenesis (Shigemoto-Mogami et al., 2014). Together, our findings support the expanding appreciation for roles of classically defined inflammatory cells in patterning and re-establishment of homeostasis after natural perturbation of neural tissue structure. This emerging role of inflammatory cells in maintaining neural homeostasis highlights the need for the field to shift from discussing the roles of glia and neural macrophages in overly general terms of neuroinflammation toward reference to the specific signaling mechanisms and behaviors of these cells.

## Synthesis

Reviewing Editor: Margaret McCarthy, Univ of Maryland School of Medicine Decisions are customarily a result of the Reviewing Editor and the peer reviewers coming together and discussing their recommendations until a consensus is reached. When revisions are invited, a fact-based synthesis statement explaining their decision and outlining what is needed to prepare a revision will be listed below. The following reviewer(s) agreed to reveal their identity: Charlotte Boettiger. Note: If this manuscript was transferred from JNeurosci and a decision was made to accept the manuscript without peer review, a brief statement to this effect will instead be what is listed below.

This revised manuscript reports the novel finding that the anti-inflammatory drug, minocycline, blocks neurogenesis in response to natural, seasonal neuron loss in an avian song nucleus. Moreover, they find that local injection of endotoxin in this nucleus promotes cell proliferation in the ventricular zone supplying this nucleus with new neurons. This collection of studies contributes to evidence linking adult neurogenesis and inflammation and establish the avian song circuit as a useful model system for further investigations in this area. The results are interesting, although some of the statistical reporting remains confusing. Below, I detail my criticisms and suggestions for improvement.

Major Comments

1. The statistical results reported in Tables 1 and 2 are confusing. First, for Table one, four columns of statistical results are included: “two-way; main effect", “Injection Type", “Condition", and “Injection*Condition.” Presumably, columns 2 and 3 represent the indicate the main effect of each factor, and the last column indicates the interaction effect, but the first column is puzzling and not meaningful.

2. Moreover, rather than simply reporting p-values for the main and interacting effects, it would be useful if the full F-statistics were reported.

3. Superscripts are also used but not defined.

4. Similarly, Table 2 includes a single column of statistical results (“ANOVA (one-way)”), but multiple factors are included in the table. Based on the text, it would seem that the statistical results reflect the effect of minocycline, but the F-values, suggest that a two factor ANOVA was carried out, although without a LDT-vehicle group, that would not be possible.

5. Although not required, we are encouraging authors to include effect sizes when the data are appropriate, as is the case here.

Minor comments:

1. Both reviewers stated that the Title is vague. It is appreciated that you would like to retain the term “inflammation” so I suggest “Inflammation induced by LPS treatment... “ or something similar.

2. LPS induces aromatase in the songbird brain. This paper should be cited and mentioned in the Discussion.

3. The first paragraph of the Methods describing the age of the animals hard to follow. Are the authors trying to say that the animals were all between 2-14 months old? If so, perhaps you could just state that more clearly.

4. The sentence on page 6 spanning lines 110-116 (marked up copy), seems to be missing “as detected” before “with Nissl.”

5. There are a few places throughout the manuscript where the authors use the term “transferred” to refer to the change in light-cycle, which erroneously suggests physical transport of the birds. I recommend replacing all of these instances to “transitioned” to avid confusion.

6. In the description of the two-way ANOVA used (line 221, marked copy), “as factors” should replace “as the factor.”

7. On line 355, the authors refer to “Hu positive HVC neurons,” which could use some explanation to the general audience of eNeuro.

8. There is a typo on line 387 (marked copy); it should read “...to the increase induced by cell death...”.

9. The sentence spanning lines 418-419reads a bit awkwardly and should be rephrased.
